# Correlation between biopterin levels and intimal-media thickness in type-2 diabetic hypertensive patients

**DOI:** 10.1186/2251-6581-13-6

**Published:** 2014-01-06

**Authors:** Alberto Francisco Rubio-Guerra, Hilda Vargas-Robles, Leonardo Del Valle-Mondragon, Alberto Maceda-Serrano, Saul Huerta-Ramirez, Montserrat Berenice Duran-Salgado, Bruno Alfonso Escalante-Acosta

**Affiliations:** 1 Hospital General de Ticomán SS and Mexican Group for Basic And Clinical Research in Internal Medicine, México, DF C.P. 03600, Mexico; 2 Centro de Investigación y de Estudios Avanzados del IPN, México, DF, Mexico; 3 Departamento de Farmacología, Instituto Nacional de Cardiología “Ignacio Chávez”, México, Mexico; 4 Centro de Investigación y de Estudios Avanzados del IPN, Monterrey, NL, Mexico

**Keywords:** Tetrahydrobiopterin, Dihydrobiopterin, BH4/BH2 ratio. intima-media thickness, Endothelial dysfunction

## Abstract

**Background:**

Biopterins have a crucial role in the function of nitric oxide synthase, uncoupling of the enzyme leads to endothelial dysfunction and vascular damage, The aim of this study was to evaluate the relationship between the levels of biopterins with carotid intima-media thickness (CIMT) in hypertensive type-2 diabetic patients.

**Methods:**

We studied 30 hypertensive type-2 diabetic patients and 30 normotensive non-diabetic age-matched subjects, in whom biopterins levels were measured by reverse phase high performance liquid chromatography with fluorescence detection. Additionally, the CIMT of both the common and internal carotid arteries was measured. The levels of biopterins and CIMT were correlated using the Pearson correlation coefficient test.

**Results:**

We did not find a significantly correlation between biopterins levels and CIMT. However, we found a significantly inverse correlation between the BH4/BH2 ratio and the CIMT in patients (r = -0.54, p < 0.01). A multiple regression analysis revealed that the CIMT correlated significantly and independently with the BH4/BH2 ratio.

**Conclusion:**

Our results suggest that the BH4/BH2 ratio seems to be a better marker of vascular disease than biopterin levels.

## Background

The endothelium maintains the integrity of vascular system, controlling vascular relaxation and contraction, the mechanism is the interaction between nitric oxide and the vasoconstrictor factors [1].

**Figure 1:**
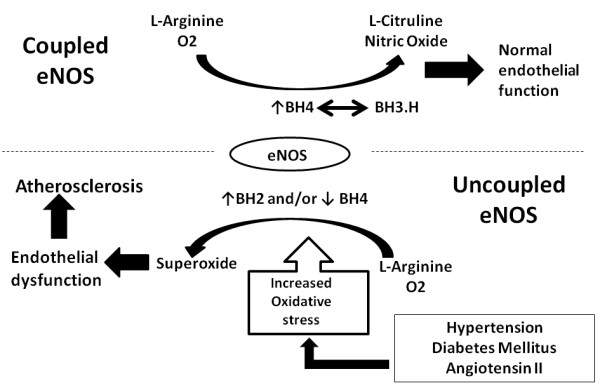
Role of biopterins in normal endothelial function and in endothelial dysfunction.

Tetrahydrobiopterin (BH4) is an essential cofactor for the nitric oxide synthase (eNOS) regulation; BH4 is a significant determinant of nitric oxide (NO) bioavailability [2]. When BH4 is oxidized to dihydrobiopterin (BH2), the bioavailability of BH4 for eNOS is reduced, leading to endothelial dysfunction, inflammation and atherosclerosis (Figure 1) [1]. Interestingly, Noguchi et als found that increased BH2 levels causes endothelial dysfunction even in the absence of BH4 deficiency in rats in vivo, through eNOS uncoupling [3].

Recent studies show that BH4/BH2 ratio may be even more important than the absolute BH4 levels for an adequate nitric oxide mediated endothelial function [4].

The increase in the thickness of the carotid intima-media measured by high resolution ultrasonography, has been directly associated with atherosclerosis, myocardial infarction and stroke, high-resolution B-mode ultrasonography provides a noninvasive method that can detect an increase in arterial wall thickness and may be used as a screening test for the evaluation of the risk of coronary artery disease in diabetic patients [5].

Although the role of BH4 in the maintenance of endothelial function is well understood, few data is available about the association between the thickness of the carotid intima-media (CIMT) and the levels of biopterins [1].

The aim of this study was to evaluate if there is a correlation between the circulating levels of biopterins, with the CIMT in hypertensive type-2 diabetic patients.

## Materials and methods

A total of 30 hypertensive patients with type-2 diabetes mellitus (> 12 months) , referred from primary care clinics, were included in this study, all of them were thiazolindinedione-naive, statin-naive and ACE/ARB-naïve. A control group of 30 normotensive non-diabetic age-matched subjects was also included.

In all of them blood pressure and body mass index were registered, blood pressure was measured three times using an aneroid sphygmomanometer (Welch Allyn NY. USA) in the sitting position after a 5-minute rest, each blood pressure measurement consisted in 3 readings of blood pressure 3 minutes apart; an average of the three measurements was recorded. Whereas Body mass index (BMI) was calculated with the next formula:

$$\frac{\text{Weight}\;\left(\mathrm{kg}\right)}{{\left[\text{Height}\;\left(\text{meters}\right)\right]}^{2}}$$

In all subjects, biopterins were measured by reverse phase high performance liquid chromatography with fluorescence detection, all venous samples were collected in the morning after a 12-h overnight fast. Also, fasting glycemia (glucose oxidase), and HbA1c were measured.

All patients had lipid profiles following a 12-hour fast by the CHOP enzymatic method, low density lipoproteins (LDL) values were calculated using Friedewald’s method.

B-mode color imaging of extracranial carotid arteries was obtained using high-resolution ultrasound (ESAUTE MEGAGP, Italia) equipped with a 10 MHz linear transducer. Subjects were evaluated lying in the supine position with hyperextension of the neck. Measurements of the distal wall of both; the common and internal carotid arteries were obtained. Registers were performed at the end of the diastole, and all determinations were performed by the same certified ultrasonographer that was blinded to the study.

Patients with any of the following diagnoses were excluded from the study:

Decompensated diabetes mellitus (fasting blood glucose >13.87 mmol/L); heart, hepatic, or renal failure; evidence of valvular heart disease; heart block or cardiac arrhythmia; acute coronary syndrome (myocardial infarction, coronary artery disease) or cerebrovascular disease six months before the study’s initiation; autoimmune disease; pregnancy; urinary tract infection; fever; or a history of alcohol abuse and/or psychotropic drugs.

Statistical analysis:

Data is presented as the mean ± standard deviation (absolute values). The relationship between the levels of biopterins and the CIMT was assessed by the Pearson correlation coefficient test.

To further investigate whether BH4/BH2 ratio can explain the CIMT independent of the other factors, multiple linear regression analysis was performed.

The study was conducted with the approval of the Research and Medical Ethics Committee of our hospital, in accordance with the Helsinki declaration. Participants provided informed, written consent before their inclusion in the study protocol.

## Results

Baseline characteristics of patients and controls are shown in Table 1. Briefly, BH2 levels were higher, and BH4 values and the BH4/BH2 ratio lower in the hypertensive type-2 diabetic patients, compared to controls.

**Table 1:** Basal characteristic of patients

**Variable; mean ± SD**	**DM2 hypertensive patients**	**Control group**	**p**
Sex (Male/Female)	16/14	19/11	ns
Glycemia (mmol/L)	7.31 ± 1.59	5.5 ± 0.1	<0.001
Glycated hemoglobin (%)	6	5	P < 0.01
LDL (mmol/L)	3.25 ± 0.75	3.22 ± 0.70	Ns
Systolic blood pressure (mm Hg)	175 ± 16	122 ± 10	<0.001
Diastolic lood pressure (mm Hg)	93 ± 8.8	76 ± 4	<0.01
Body Mass Index	30.4 ± 5	29.8 ± 6	Ns
BH4 (nmol/L)	5.23 ± 1.33	7.07 ± 1.34	P < 0.05
BH2 (nmol/L)	9.97 ± 2.6	7.56 ± 1.57	P < 0.05
BH4/BH2 Ratio	0.701 ± 0.14	1.34 ± 0.16	P < 0.01
Carotid Intima/Media thickness (mm)	1.43 ± 0.43	0.33 ± 0.06	P < 0.005
History of DM2	8.36 years		
History of Hypertension	11.3 years		

*LDL*, low density lipoprotein.

BH4 = Tetrahydrobiopterin.

BH2 = Dihydrobiopterin.

**Figure 2:**
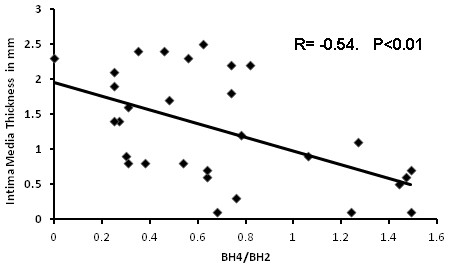
Correlation between the BH4/BH2 ratio and carotidal intima-media thickness in hypertensive type-2 diabetic patients.

In patients we were unable to demonstrate any relationship between CIMT and BH4 (r = -0.044, p > 0.1), nor with BH2 (r = 0.077, p > 0.1). ). Whereas the BH4/BH2 ratio correlated negatively with CMIT (r = - 0.54, p < 0.01) (Figure 2).

In the control group no correlation was observed between CIMT and BH4 (r = -0.158, p > 0.05), BH2 (r = 0.27, p > 0.05), nor with the BH4/BH2 ratio (r = - 0.28, p > 0.05).

The results of the Pearson correlation analysis comparing CIMT with other risk factors are listed in Table 2, CIMT correlated positively with low density lipoprotein, systolic blood pressure, HbA1C, body mass index, and with the duration of both; diabetes mellitus and hypertension, but not with age, nor with diastolic blood pressure.

**Table 2:** Relationship between (CIMT) and various risk factors in diabetic hypertensive patients

	**Pearson correlation coefficient**	
**Characteristics**	**r**	**P**
BH4/BH2 Ratio	0.54	<0.01
LDL Cholesterol	0.46	<0.01
HbA1C	0.42	<0.05
Duration of Diabetes	0.42	<0.05
Systolic blood pressure	0.41	<0.05
Duration of Hypertension	0.39	<0.05
Body Mass Index	0.38	<0.05
Diastolic Blood Pressure	0.35	>0.05
Age	0.17	>0.05

*NS*, not significantly.

*LDL*, low density lipoprotein.

Multiple regression analysis revealed that the CIMT correlated significantly and independently with the BH4/BH2 ratio (Table 3).

**Table 3:** Multivariate analysis using multiple linear regression

	**Multiple linear regression**
**Variable**	**p**
BH4/BH2 ratio	<0.01
Systolic blood pressure	<0.05
LDL	>0.05

*LDL*, low density lipoprotein.

Multivariate model with 3 factors. LDL does not reach significance, and only BH4/BH2 ratio and systolic blood pressure were independently associated with the CIMT.

## Discussion

In this study, circulating levels of BH4 and BH2 did not correlate with the CIMT in hypertensive type-2 diabetic patients, nor in controls, whereas a significant association was observed for the BH4/BH2 ratio with CIMT in patients, but not in controls. It is also relevant to say that our patients were ACE/ARB-naïve and statin-naïve because those drugs have been shown to raise circulating levels of BH4 and to decrease those of BH2 [1]. It is assumed that plasma levels of biopterins reflect their endothelial levels, then; the plasma BH4/BH2 ratio can serve as a reliable marker of oxidative stress on the endothelium [4].

We observed that CIMT was significantly greater in diabetic patients that in controls, this fact agree with the findings of Mansouri et als [5], and is not surprising since atherosclerosis is the main cause of mortality in diabetic patients [6], we also found that CIMT correlated positively with the levels of LDL, HbA1C, systolic blood pressure, and body mass index, and also with duration of both, type 2 diabetes and hypertension, but not with age and diastolic blood pressure, our results agree with those of Kotb et als; they have reported that in obese diabetic adolescents; diabetes duration, body mass index and systolic blood pressure were the main determinants of CIMT [7].

A number of studies implicate BH4 in the regulation of vascular function. In the absence of enough BH4, instead of oxidizing L-arginine, eNOS reduces molecular oxygen to superoxide, leading to endothelial dysfunction, when nitric oxide reacts with superoxide lost its vasodilator and antiatherogenic effects (Figure 1) [1,3]. Then, an inverse relation between BH4 and the development of vascular damage and atherosclerotic plaque progression is expected; although in our work, the association between BH4 levels with intima-media thickness did not reach significance.

Several studies have shown that when BH4 is oxidized to BH2, the bioavailability of BH4 for eNOS is reduced. Besides, BH2 (which has no cofactor activity) may compete with BH4 for the oxygenase domain in eNOS, leading to a decreased eNOS activity, endothelial dysfunction and vascular disease [8], previous studies have shown that increased BH2 levels cause endothelial dysfunction through eNOS uncoupling in rats (Figure 1), even in the absence of BH4 deficiency [3]. However, in our paper we found that patients and controls shown a non-significant association between BH2 levels and intima-media thickness.

It has been proposed that BH4/BH2 ratio may be even more important than the absolute BH4 value for eNOS functionality [4]. As this ratio includes information about the levels of both biopterins, may be a more sensitive and reliable marker of CIMT than the isolate values of BH4 or BH2, with a higher BH4/BH2 ratio suggesting an adequate eNOS bioactivity, Our results appear to support this idea since we found a significantly inverse correlation between the BH4/BH2 ratio and intima-media thickness in patients. This correlation was significant and independent of other risk factors as LDL and systolic blood pressure values

In the control group we did not find any correlation between the CIMT and none of the parameters evaluated; this must be due to the fact that CIMT in this group was almost normal.

In a previous paper we have reported an inverse association between the systemic levels of adhesion molecules with the BH4/BH2 ratio in type 2 diabetic patients [9], since the levels of adhesion molecules correlated with the CIMT in those patients [10]; our findings enforce the growing body of research establishing the association between the BH4/BH2 ratio and vascular disease.

Our results may have therapeutics implications; several in vivo studies in humans have shown the beneficial effect of BH4 on endothelial dysfunction and its vasoprotective properties. Then, enhancing BH4 synthesis or bioavailability in endothelial cells may be an adequate strategy for the prevention and treatment of cardiovascular diseases, especially in high risk patients [11]. Interestingly, Oueda reported that oral administration of BH4 restored endothelial function in smokers, -whereas tetrahydroneopterin, which has the antioxidant capability of BH4, but not its cofactor activity, had no effect- [12], suggesting restoration of the eNOS bioactivity as the underlying mechanism.

The use of drugs that increase the BH4/BH2 ratio-, as statins, also prevent the vascular complications in type-2 diabetic patients, not only by their effects on low density cholesterol, but also via cholesterol-independent effects, and their action on biopterin levels may be implicated, this fact requires further research [13].

## Conclusion

Our results shown that a low BH4/BH2 ratio, instead of BH4 or BH2 levels, may be a marker of vascular disease, independent of other risk factors such LDL and HA1C levels, hypertension, age and body mass index. Estimation of BH4/BH2 ratio may improve individual risk assessment.

## Competing interests

All the authors declare that they have no competing interests, this research received no specific grant from any funding agency in the public, commercial, or not-for-profit sectors.

## Authors’ contributions

AFRG: Conception, design, analysis and interpretation of results. HVR: Acquisition and analysis of data. LDVM: Acquisition and analysis of data. AMS. Acquisition of data. SHR, Analysis and interpretation of data, added data and their interpretation. MBDS. Study researcher, interpretation of results. BAEA. Study researcher, interpretation of results. All authors have contributed to, seen and approved the manuscript.
